# Draft Genome Sequences of Aeromonas schubertii Strains Isolated from Asian Seabass from Thailand

**DOI:** 10.1128/mra.01007-21

**Published:** 2022-01-06

**Authors:** Komkiew Pinpimai, Kitipong Angsujinda, Tongchai Thitiphuree, Sirikorn Kitiyodom, Putita Chokmangmeepisarn, Nopadon Pirarat

**Affiliations:** a Aquatic Resources Research Institute, Chulalongkorn University, Bangkok, Thailand; b Department of Pathology, Faculty of Veterinary Science, Chulalongkorn University, Bangkok, Thailand; c Center of Excellence in Fish Infectious Diseases, Department of Veterinary Microbiology, Faculty of Veterinary Science, Chulalongkorn University, Bangkok, Thailand; University of Maryland School of Medicine

## Abstract

Aeromonas schubertii is a Gram-negative, rod-shaped bacterium. It is a rare species that has been reported in humans and aquatic animals. Here, we report the genome sequences of A. schubertii strains isolated from two mass mortality events in central Thailand that were associated with aquaculture of Asian seabass.

## ANNOUNCEMENT

*Aeromonas* species are ubiquitous in aquatic environments (1). They cause a variety of infections in aquatic animals and humans (2). In aquatic animals, Aeromonas hydrophila, Aeromonas veronii, and Aeromonas salmonicida are mostly reported as the causes of significant deaths in a number of fish species (1). In this decade, there has been an increase in other species such as Aeromonas schubertii, which was reported as the cause of death in several fish species, including snakehead fish (3), tilapia (4), and zebra fish (5). A. schubertii can also be isolated from frogs (6), shrimp (7), and mussels (8). Here, we report the draft whole-genome sequences of A. schubertii strains that were isolated from Asian seabass (Lates calcarifer) from two outbreak events.

A. schubertii CHULA2021a was isolated from Asian seabass from an outbreak in November 2020 in a commercial farm in central Thailand, with a mortality rate of over 80%. In June 2021, A. schubertii CHULA2021b was isolated from moribund and dead juvenile Asian seabass in a private laboratory unit in central Thailand, which had a mortality rate of >90%. Briefly, the trunk kidney was inoculated onto blood agar and incubated at 37°C for 24 h under aerobic conditions. If a single colony type or a mixed culture with a predominance (>80%) of one colony type was seen, then the colony was subcultured on blood agar for further identification. The bacteria were confirmed as A. schubertii using matrix-assisted laser desorption ionization–time of flight mass spectrometry (MALDI-TOF MS) (Bruker Daltonics, Billerica, MA). A single A. schubertii isolate was sequenced from each event. The PureLink genomic DNA minikit (Invitrogen, Carlsbad, CA) was used to extract genome-quality DNA from a single colony from each event that had been cultured on blood agar at 37°C for 24 h under aerobic conditions, and the DNA was sent to Macrogen, Inc. (Seoul, South Korea), for whole-genome sequencing. A fragment library was prepared using a TruSeq Nano DNA library preparation kit (Illumina, Inc., San Diego, CA). Paired-end reads (2 × 100 bp) were obtained with a HiSeq instrument (Illumina). Default parameters were used for all software unless otherwise specified. FastQC v0.11.3 (9) was used to check the quality of the data. The sample was *de novo* assembled using SPAdes v3.14.1 (10) (in careful mode). The isolate contigs produced by SPAdes were annotated by Prokka v1.14.5 (11). Genome sizes, total numbers of reads, numbers of contigs, fold coverage, *N*_50_ values, GC contents, and numbers of rRNAs, tRNAs, and transfer-messenger RNAs (tmRNAs) are summarized in Table 1.

**TABLE 1:** Description of A. schubertii strains sequenced and their genomic characteristics

Parameter	Data for:	
	Aeromonas schubertii CHULA2021a	Aeromonas schubertii CHULA2021b
GenBank accession no.	JAIRBT000000000	JAIRBS000000000
SRA accession no.	SRR15801989	SRR15801988
Total no. of reads	6,043,754	10,842,070
Genome length (bp)	4,281,475	4,225,692
Fold coverage (×)	137	264
*N*_50_ (bp)	126,401	167,967
No. of contigs	87	41
GC content (%)	61.65	61.87
No. of rRNAs	3	4
No. of tRNAs	93	83
No. of tmRNAs	1	2

Here, we report the draft genome sequences of A. schubertii strains isolated from Asian seabass. The data from this study will provide information on the genome of A. schubertii and inform future research on the role of A. schubertii infection in Asian seabass.

### Data availability.

The whole-genome shotgun sequences described here have been deposited in DDBJ/ENA/GenBank under BioProject number PRJNA761361 with accession numbers JAIRBT000000000 and JAIRBS000000000. The raw sequencing reads have been deposited in the NCBI Sequence Read Archive (SRA) under accession numbers SRR15801989 and SRR15801988.
